# Differential Effects of Hypoxia versus Hyperoxia or Physoxia on Phenotype and Energy Metabolism in Human Chondrocytes from Osteoarthritic Compared to Macroscopically Normal Cartilage

**DOI:** 10.3390/ijms24087532

**Published:** 2023-04-19

**Authors:** Lekha Jain, Scott M. Bolam, A. Paul Monk, Jacob T. Munro, Even Chen, Jade Tamatea, Nicola Dalbeth, Raewyn C. Poulsen

**Affiliations:** 1 Department of Pharmacology and Clinical Pharmacology, University of Auckland, Auckland 1023, New Zealand; l.jain@auckland.ac.nz (L.J.);; 2 Department of Surgery, University of Auckland, Auckland 1023, New Zealand; 3 Department of Medicine, University of Auckland, Auckland 1023, New Zealand; n.dalbeth@auckland.ac.nz; 4 Auckland Bioengineering Institute, University of Auckland, Auckland 1010, New Zealand; 5 Te Kupenga Hauora Māori, University of Auckland, Auckland 1010, New Zealand; j.tamatea@auckland.ac.nz

**Keywords:** glycolysis, oxidative phosphorylation, hypoxia, physoxia, chondrocyte, osteoarthritis, MMP13, aggrecan, SOX9, type II collagen

## Abstract

Chondrocyte phenotype and energy metabolism are altered in osteoarthritis (OA). However, most studies characterising the change in human chondrocyte behaviour in OA have been conducted in supraphysiological oxygen concentrations. The purpose of this study was to compare phenotype and energy metabolism in chondrocytes from macroscopically normal (MN) and OA cartilage maintained in 18.9% (standard tissue culture), 6% (equivalent to superficial zone of cartilage in vivo) or 1% oxygen (equivalent to deep zone of cartilage in vivo). MMP13 production was higher in chondrocytes from OA compared to MN cartilage in hyperoxia and physoxia but not hypoxia. Hypoxia promoted SOX9, COL2A1 and ACAN protein expression in chondrocytes from MN but not OA cartilage. OA chondrocytes used higher levels of glycolysis regardless of oxygen availability. These results show that differences in phenotype and energy metabolism between chondrocytes from OA and MN cartilage differ depending on oxygen availability. OA chondrocytes show elevated synthesis of cartilage-catabolising enzymes and chondrocytes from MN cartilage show reduced cartilage anabolism in oxygenated conditions. This is relevant as a recent study has shown that oxygen levels are elevated in OA cartilage in vivo. Our findings may indicate that this elevated cartilage oxygenation may promote cartilage loss in OA.

## 1. Introduction

Osteoarthritis (OA) is a painful, disabling disease characterised by loss of cartilage within joints [1]. In osteoarthritis, a shift in chondrocyte phenotype occurs. This is observed in both animal models as well as human disease and is implicated as a contributing factor in OA pathology [2,3,4]. In human tissue, the change in chondrocyte phenotype is specific to the osteoarthritic lesion, with distinct differences in phenotype markers between chondrocytes from this region and the adjacent macroscopically normal (MN) cartilage [5,6]. Levels of Sry-box transcription factor 9 (SOX9), the master transcriptional regulator of chondrocyte lineage determination, are lower in chondrocytes from the osteoarthritic region [7] and the SOX9-target genes *COL2A1* (collagen II) and *ACAN* (aggrecan) are also often downregulated, particularly in late-stage disease [8]. In contrast, production of cartilage-degrading enzymes such as matrix metalloproteinase 13 (MMP13) and a disintegrin and metalloproteinase with thrombospondin motif 5 (ADAMTS5) is upregulated [9,10,11,12].

This change in chondrocyte phenotype is accompanied by a change in energy metabolism [13,14,15]. Two major pathways involved in eukaryotic energy generation are glycolysis and oxidative phosphorylation (OXPHOS) [16]. These pathways are interconnected such that glucose entering the glycolytic pathway can be catabolised to pyruvate which enters the OXPHOS pathway or can continue to be metabolised to lactate [17]. Although metabolism via OXPHOS generates more ATP per unit of glucose than glycolysis, it is dependent on oxygen availability and mitochondrial activity. In contrast, glycolysis is a non-mitochondrial pathway and can occur in the presence or absence of oxygen [16]. Given cartilage is avascular and evidence of dysfunctional mitochondrial activity in OA [17], glycolysis is proposed to be the predominant pathway underlying chondrocyte energy production in disease [18,19].

Both chondrocyte phenotype and energy metabolism can differ depending on the level of oxygen exposure [17,20,21,22]. This is relevant as there is an oxygen gradient in articular cartilage. The superficial zone of human cartilage is well oxygenated by physiological standards with an estimated oxygen level of 5–7%, whereas the deep zone is hypoxic with an estimated oxygen level of 1% or lower [23]. How the phenotype and energy usage of OA and non-OA chondrocytes change with differing oxygen exposures is unclear. Understanding this is important, however, as studies with human tissue are essential for establishing the mechanisms involved in human disease yet such studies require at least some in vitro cell maintenance. The majority of previous studies comparing chondrocyte phenotype and energy metabolism between OA and non-OA chondrocytes have been conducted under oxygen conditions (19–21% O_2_) far exceeding those in cartilage in vivo [17,24,25]. Although some studies have been performed under hypoxic conditions [26,27,28], these have been performed in isolation from studies in standard tissue culture, precluding direct comparison. The purpose of this study was to directly compare phenotypic marker expression and energy metabolism pathway usage in chondrocytes from osteoarthritic and adjacent MN cartilage maintained in hyperoxic conditions (18.9% oxygen) to that in chondrocytes maintained in 6% oxygen (“physoxia”) and 1% oxygen (“hypoxia”).

## 2. Results

### 2.1. ADAMTS5 and MMP13 Expression Is Higher in Chondrocytes from Osteoarthritic Cartilage in Hyperoxia and Physoxia but Not Hypoxia

**Figure 1:**
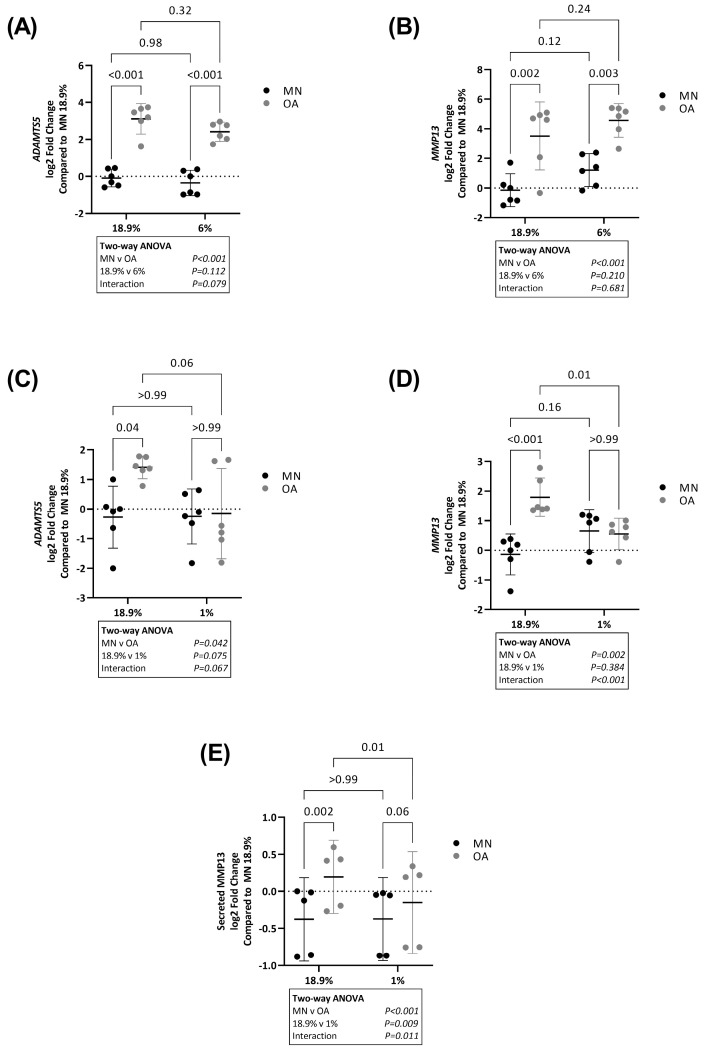
Expression of ADAMTS5 and MMP13 in OA chondrocytes and chondrocytes from macroscopically normal cartilage maintained in 18.9% compared to 6% or 1% oxygen. Paired samples of human chondrocytes isolated from macroscopically normal (MN) and osteoarthritic (OA) cartilage regions within OA joints from six patients were plated in duplicate plates. One plate was transferred to a standard tissue culture incubator (18.9% oxygen) for 24 h and the other transferred to a hypoxia incubator maintained at either 6% or 1% oxygen. Chondrocytes from a different six patients were used to compare the effects of 18.9% vs. 1% oxygen than those used to compare the effects of 18.9% vs. 6% oxygen. Comparison of RNA levels (measured by qPCR) of (**A**) ADAMTS5 and (**B**) MMP13 in cells maintained at 18.9% vs. 6% oxygen and (**C**) ADAMTS5 and (**D**) MMP13 in cells maintained at 18.9% vs. 1% oxygen. (**E**) Levels of secreted MMP13 protein as measured by ELISA in cell supernatants in chondrocytes from OA and MN cartilage maintained in 18.9% vs. 1% oxygen for 24 h (*n* = 5). Data shown are mean ± 95% CI. All data were analysed by two-way ANOVA. Statistically significant differences between groups following post hoc testing are shown on graphs. *p* < 0.05 was considered statistically significant.

To enable direct comparison of the effects of oxygen levels on chondrocyte phenotype marker expression, identical plates of chondrocytes from MN and OA cartilage from six patient donors were simultaneously cultured for 24 h in either 18.9% or 6% oxygen. Expression of *ADAMTS5* (Figure 1A) and *MMP13* (Figure 1B) was significantly higher in OA chondrocytes compared to chondrocytes from MN cartilage in both 18.9% and 6% oxygen. There was no significant difference in expression of either gene in chondrocytes from MN cartilage maintained in 18.9% oxygen compared to the same cells maintained in 6% oxygen or in OA chondrocytes maintained in 18.9% oxygen compared to the same cells maintained in 6% oxygen (Figure 1A,B).

To determine the effects of exposure to 1% oxygen, chondrocytes from MN and OA cartilage from another six patient donors were plated in duplicate plates and simultaneously maintained in either 18.9% or 1% oxygen. As before, *ADAMTS5* and *MMP13* expression was significantly higher in OA chondrocytes compared to chondrocytes from MN cartilage maintained in 18.9% oxygen but there was no difference in expression of either gene between chondrocytes from the two tissue regions when the same cells were maintained in 1% oxygen (Figure 1C,D). Expression of both genes was similar in chondrocytes from MN cartilage maintained in 18.9% oxygen compared to 1% oxygen (Figure 1C,D). Heterogeneity was observed between patient donors in *ADAMTS5* expression in OA chondrocytes maintained in 1% oxygen and there was also no significant difference between the 1% and 18.9% oxygen conditions in these cells (*p* = 0.06) (Figure 1C).

Both RNA levels (Figure 1D) and secreted protein levels (Figure 1E) of MMP13 were significantly lower in OA chondrocytes in 1% compared to 18.9% oxygen (Figure 1D). MMP13 protein levels were significantly higher in OA chondrocytes compared to chondrocytes from MN cartilage in 18.9% oxygen, but not 1% oxygen (Figure 1E).

### 2.2. SOX9, COL2A1 and ACAN Are Upregulated in Chondrocytes from Macroscopically Normal but Not Osteoarthritic Cartilage in Hypoxia

**Figure 2:**
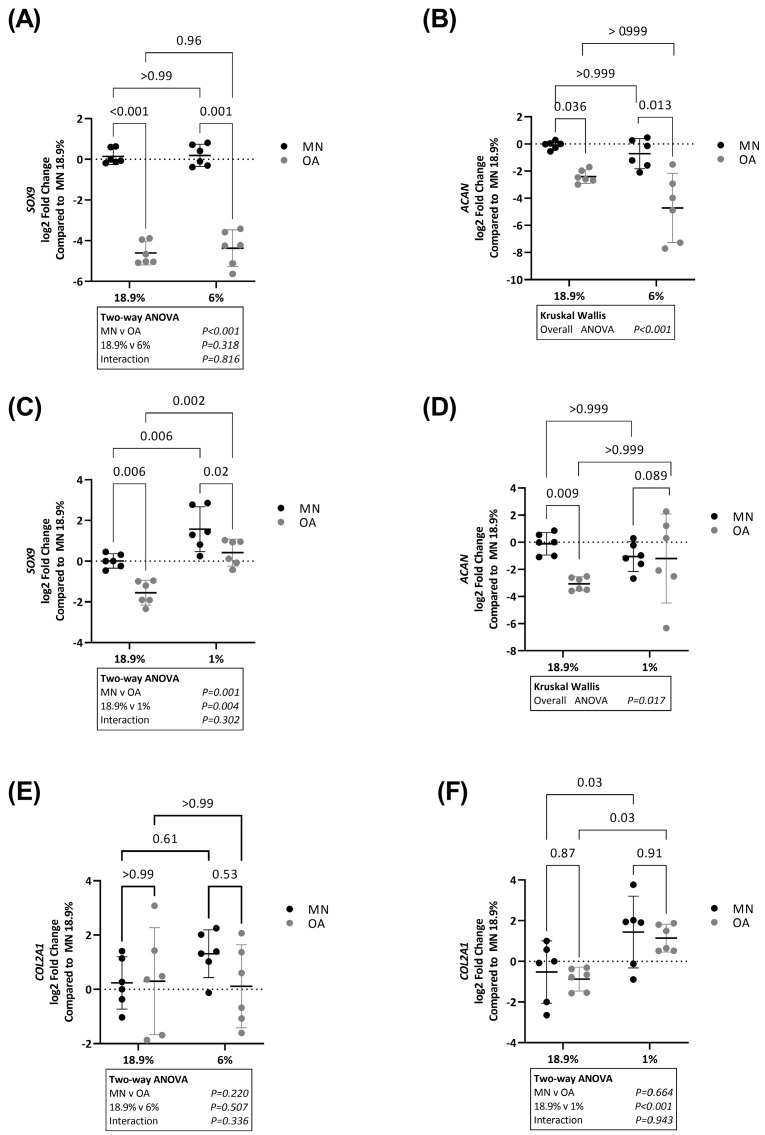
mRNA levels of SOX9, COL2A1 and ACAN, in OA chondrocytes and chondrocytes from macroscopically normal cartilage maintained in 18.9% compared to 6% or 1% oxygen. Paired samples of human chondrocytes isolated from macroscopically normal (MN) and osteoarthritic (OA) cartilage regions within OA joints from six patients were plated in duplicate plates. One plate was transferred to a standard tissue culture incubator (18.9% oxygen) and the other transferred to a hypoxia incubator maintained at either 6% or 1% oxygen for 24 h. Chondrocytes from a different six patients were used to compare the effects of 18.9% vs. 1% oxygen than those used to compare the effects of 18.9% vs. 6% oxygen. Comparison of RNA levels (measured by qPCR) of (**A**) SOX9 and (**B**) ACAN in cells maintained in 18.9% vs. 6% oxygen and (**C**) SOX9 and (**D**) ACAN in cells maintained in 18.9% vs. 1% oxygen. Expression of (**E**) COL2A1 in cells maintained in 18.9% vs. 6% oxygen and (**F**) COL2A1 in cells maintained in 18.9% vs. 1% oxygen. Data shown are mean ± 95% CI. Data for ACAN were analysed by Kruskal–Wallis test, all other data were analysed by two-way ANOVA. Statistically significant differences between groups following post hoc testing are shown on graphs. *p* < 0.05 was considered statistically significant.

Next, we compared expression of *SOX9*, *ACAN* and *COL2A1* in the same cell samples as above. *SOX9* (Figure 2A) and *ACAN* (Figure 2B) expression was lower in OA chondrocytes compared to chondrocytes from MN cartilage in 18.9% and 6% oxygen. *SOX9* levels were still significantly lower in OA chondrocytes compared to chondrocytes from MN cartilage in 1% oxygen but *SOX9* levels were overall higher in chondrocytes from both tissue regions in 1% oxygen (Figure 2C). In contrast, there was no difference in *ACAN* expression between OA chondrocytes and chondrocytes from MN cartilage when maintained in 1% oxygen (Figure 2D).

There was no significant difference in *COL2A1* expression between OA chondrocytes and chondrocytes from MN cartilage in 18.9%, 6% or 1% oxygen concentrations (Figure 2E). However, overall expression of *COL2A1* was significantly higher in chondrocytes from both tissue regions when maintained in 1% compared to 18.9% oxygen (Figure 2F).

**Figure 3:**
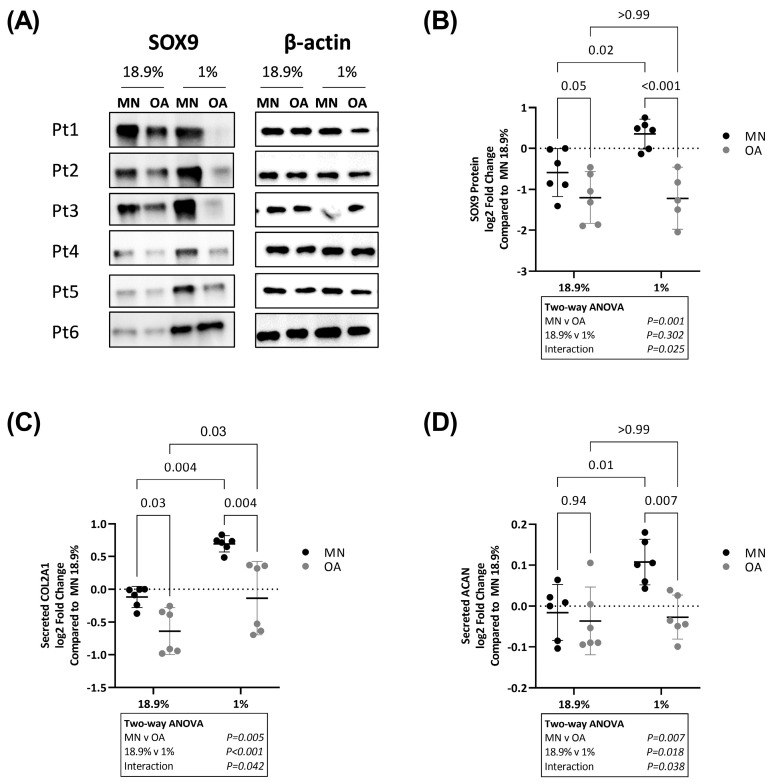
Protein levels of SOX9, COL2A1 and ACAN in OA chondrocytes and chondrocytes from macroscopically normal cartilage maintained in 18.9% compared to 6% or 1% oxygen. Paired samples of human chondrocytes isolated from macroscopically normal (MN) and osteoarthritic (OA) cartilage regions within OA joints from six patients were plated in duplicate plates. One plate was transferred to a standard tissue culture incubator (18.9% oxygen) and the other transferred to a hypoxia incubator maintained at 1% oxygen for 24 h. (**A**) Western blots showing SOX9 levels and (**B**) quantification of SOX9 protein expression using band densitometry in OA chondrocytes and chondrocytes from MN cartilage from 6 patient donors maintained at 18.9% vs. 1% oxygen. (**C**) Levels of secreted type II collagen protein and (**D**) levels of secreted ACAN protein as measured by ELISA in cell supernatants from OA chondrocytes and chondrocytes from MN cartilage maintained in 18.9% vs. 1% oxygen for 24 h. Data shown are mean ± 95% CI. All data were analysed by two-way ANOVA. Statistically significant differences between groups following post hoc testing are shown on graphs. *p* < 0.05 was considered statistically significant.

We further assessed whether these differences in *SOX9*, *COL2A1* and *ACAN* gene expression were also apparent at the protein level. By Western blot, protein levels of SOX9 were lower in OA chondrocytes compared to chondrocytes from MN cartilage when cells were maintained in either 18.9% oxygen or 1% oxygen (Figure 3A,B). There was evidence that OA chondrocytes and chondrocytes from MN cartilage responded differently to varying oxygen levels (significant interaction on two-way ANOVA, *p* = 0.025). By post hoc analysis, SOX9 protein levels were significantly higher in chondrocytes from MN cartilage maintained in 1% compared to 18.9% oxygen but the same was not observed for OA chondrocytes (Figure 3A,B). Similarly, protein levels of secreted COL2A1 and ACAN were higher in chondrocytes from MN cartilage maintained in 1% compared to 18.9% oxygen (Figure 3C,D). There was no significant difference in levels of either protein between chondrocytes from the two cartilage regions in 18.9% oxygen, but both proteins were lower in OA chondrocytes compared to chondrocytes from MN cartilage when cells were maintained in 1% oxygen (Figure 3C,D).

### 2.3. Glucose Consumption Is Higher in Chondrocytes from Osteoarthritic Cartilage in Hyperoxia, Physoxia and Hypoxia

**Figure 4:**
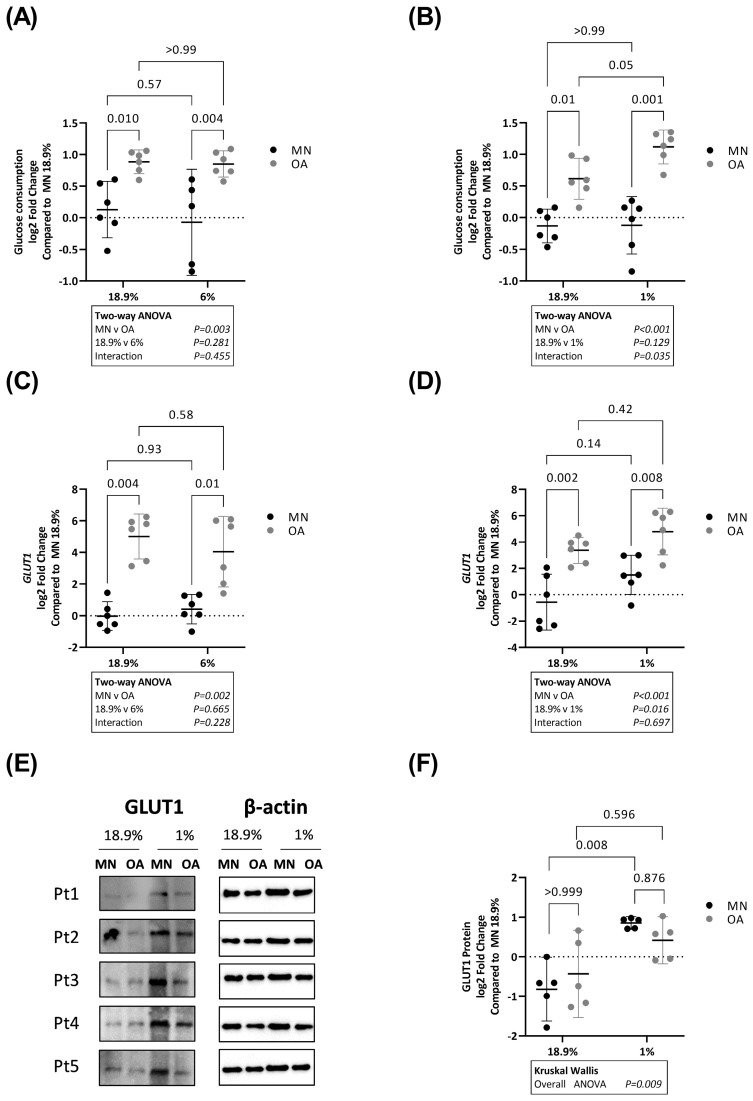
Glucose consumption and GLUT1 glucose transporter levels in OA chondrocytes and chondrocytes from macroscopically normal cartilage maintained in 18.9% compared to 6% or 1% oxygen. Paired samples of human chondrocytes isolated from macroscopically normal (MN) and osteoarthritic (OA) cartilage regions within OA joints from six patients were plated in duplicate plates. One plate was transferred to a standard tissue culture incubator (18.9% oxygen) and the other transferred to a hypoxia incubator maintained at either 6% or 1% oxygen for 24 h. Chondrocytes from a different six patients were used to compare the effects of 18.9% vs. 1% oxygen than those used to compare the effects of 18.9% vs. 6% oxygen. Comparison of glucose consumption in (**A**) OA chondrocytes and chondrocytes from MN cartilage maintained in 18.9% vs. 6% oxygen and (**B**) OA chondrocytes and chondrocytes from MN cartilage maintained in 18.9% vs. 1% oxygen. Comparison of RNA levels (measured by qPCR) of (**C**) GLUT1 in cells maintained in 18.9% vs. 6% oxygen and (**D**) GLUT1 in cells maintained in 18.9% vs. 1% oxygen. (**E**) Western blots showing GLUT1 protein levels and (**F**) quantification of GLUT1 protein expression using band densitometry in OA chondrocytes and chondrocytes from MN cartilage maintained in 18.9% vs. 1% oxygen (*n* = 5). Pt = patient. Data shown are mean ± 95% CI. GLUT1 Western blot data were analysed by Kruskal–Wallis test. All other data were analysed by two-way ANOVA. Statistically significant differences between groups following post hoc testing are shown on graphs. *p* < 0.05 was considered statistically significant.

Using the same experimental strategy as above, glucose consumption was directly compared in OA chondrocytes and chondrocytes from MN cartilage maintained in 18.9% vs. 6% oxygen and 18.9% vs. 1% oxygen. Glucose consumption was significantly higher in OA chondrocytes compared to chondrocytes from MN cartilage at 18.9% oxygen (Figure 4A). This difference was also evident at both 6% and 1% oxygen (Figure 4A,B). There was no difference in glucose consumption between chondrocytes from MN cartilage maintained in 18.9% vs. 6% oxygen. Similarly, there was no difference in glucose consumption in OA chondrocytes maintained in 18.9% vs. 6% oxygen. However, there was evidence that chondrocytes from the two cartilage regions responded differently to 1% oxygen (significant interaction on two-way ANOVA (*p* = 0.035)) (Figure 4B). While glucose consumption was similar in chondrocytes from MN cartilage maintained in 18.9% vs. 1% oxygen, it was higher in OA chondrocytes maintained at 1% vs. 18.9% oxygen.

*GLUT1* RNA levels were higher in OA chondrocytes compared to chondrocytes from MN cartilage under all three oxygen levels (Figure 4C,D). There was no difference in *GLUT1* expression between chondrocytes from MN cartilage maintained in 18.9% oxygen compared to the same cells maintained in 6% oxygen. Similarly, there was no difference in *GLUT1* expression in OA chondrocytes maintained in 18.9% oxygen compared to the same cells maintained in 6% oxygen. However, overall RNA levels of *GLUT1* were higher in 1% oxygen compared to 18.9% oxygen (significant main effect of 18.9% vs. 1% on two-way ANOVA, *p* = 0.016). In contrast, there was no significant difference in GLUT1 protein levels between chondrocytes from the two cartilage regions in either 18.9% or 1% oxygen, but GLUT1 protein levels were significantly higher in chondrocytes from MN cartilage in 1% oxygen compared to the same cells in 18.9% oxygen (Figure 4F).

### 2.4. Chondrocytes from Osteoarthritic Cartilage Utilise More Glycolysis Than Chondrocytes from Macroscopically Normal Cartilage in Hyperoxia, Physoxia and Hypoxia

**Figure 5:**
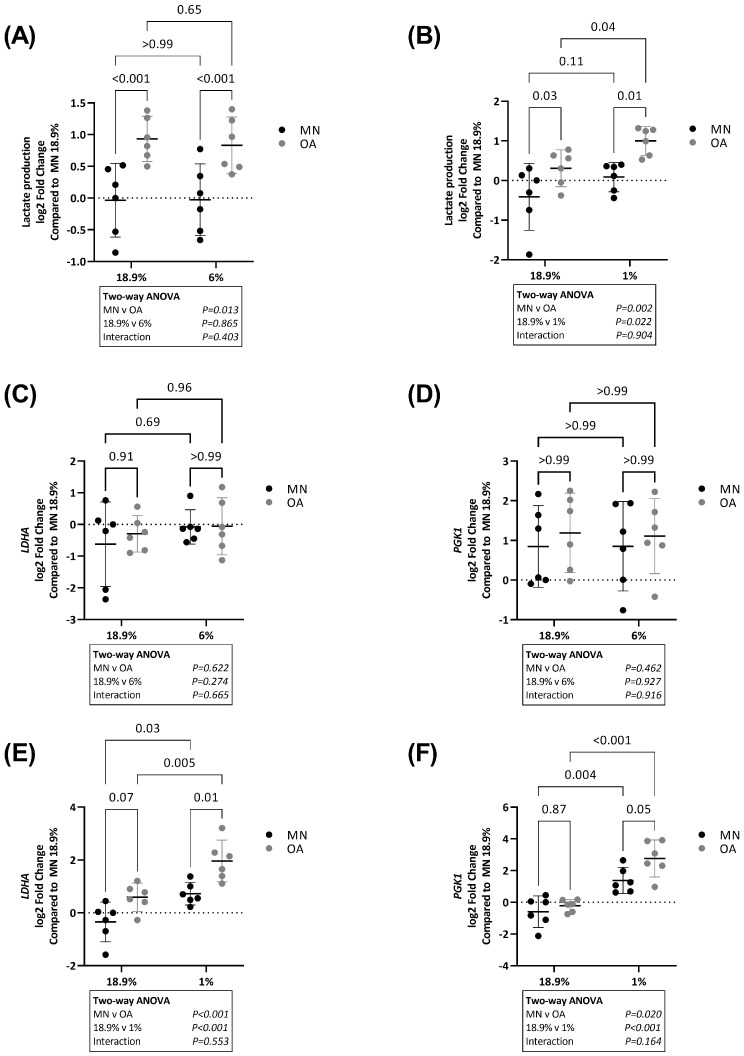
Lactate production and expression of the glycolytic genes LDHA and PGK1 in OA chondrocytes and chondrocytes from macroscopically normal cartilage maintained in 18.9% compared to 6% or 1% oxygen. Paired samples of human chondrocytes isolated from macroscopically normal (MN) and osteoarthritic (OA) cartilage regions within OA joints from six patients were plated in duplicate plates. One plate was transferred to a standard tissue culture incubator (18.9% oxygen) and the other transferred to a hypoxia incubator maintained at either 6% or 1% oxygen for 24 h. Chondrocytes from a different six patients were used to compare the effects of 18.9% vs. 1% oxygen than those used to compare the effects of 18.9% vs. 6% oxygen. Comparison of lactate production in (**A**) cells maintained in 18.9% vs. 6% oxygen and (**B**) cells maintained in 18.9% vs. 1% oxygen. Comparison of RNA levels (measured by qPCR) of (**C**) LDHA and (**D**) PGK1 in cells maintained in 18.9% vs. 6% oxygen and (**E**) LDHA and (**F**) PGK1 in cells maintained in 18.9% vs. 1% oxygen. Data shown are mean ± 95% CI. All data were analysed by two-way ANOVA. Statistically significant differences between groups following post hoc testing are shown on graphs. *p* < 0.05 was considered statistically significant.

Lactate production was higher in OA chondrocytes compared to chondrocytes from MN cartilage in 18.9%, 6% and 1% oxygen (Figure 5A,B). There was no significant difference in the level of lactate production between chondrocytes from MN cartilage in 6% vs. 18.9% oxygen or 1% vs. 18.9% oxygen. Lactate production was not significantly different between OA chondrocytes in 6% vs. 18.9% oxygen but lactate production was higher in OA chondrocytes in 1% vs. 18.9% oxygen (Figure 5B).

Next, we compared the expression of glycolytic genes between chondrocytes from OA and MN cartilage maintained under different oxygen conditions. There was no difference in expression of lactate dehydrogenase (*LDHA*) or phosphoglycerate kinase 1 (*PGK1*) between chondrocytes from the two cartilage regions in either 18.9% or 6% oxygen (Figure 5C,D). However, RNA levels of *LDHA* and *PGK1* were significantly higher in OA chondrocytes compared to chondrocytes from MN cartilage in 1% oxygen (Figure 5E,F). Expression of *LDHA* and *PGK1* was overall higher in chondrocytes from both cartilage regions when maintained in 1% compared to 18.9% oxygen (Figure 5E,F).

**Figure 6:**
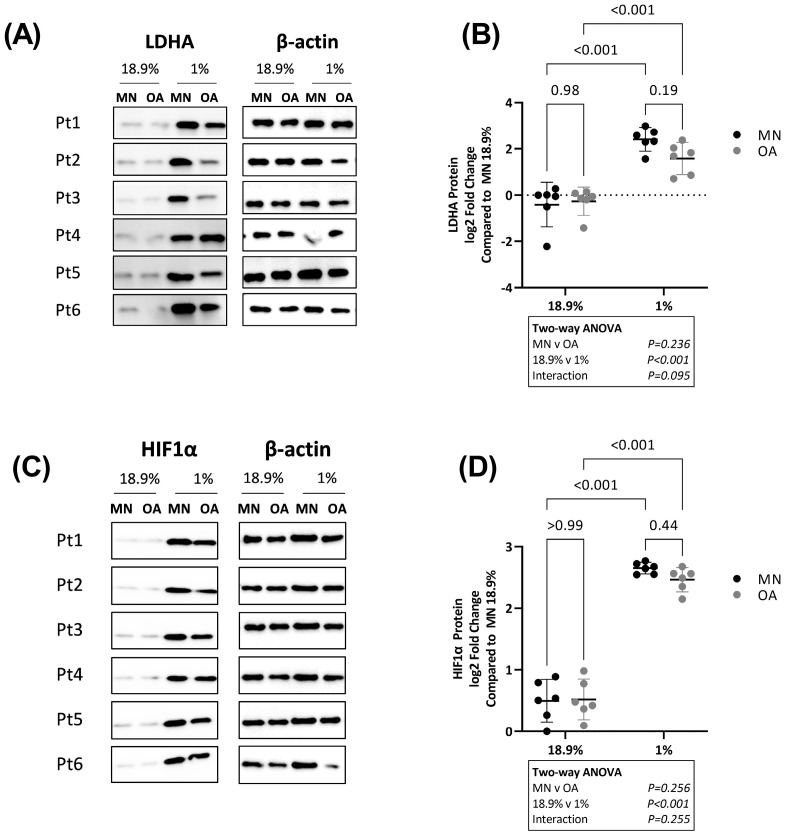
Protein levels of LDHA and HIF1α in OA chondrocytes and chondrocytes from macroscopically normal cartilage maintained in 18.9% compared to 6% or 1% oxygen. Paired samples of human chondrocytes isolated from macroscopically normal (MN) and osteoarthritic (OA) cartilage regions within OA joints from six patients were plated in duplicate plates. One plate was transferred to a standard tissue culture incubator (18.9% oxygen) and the other transferred to a hypoxia incubator maintained at 1% oxygen for 24 h. (**A**) Western blots for LDHA and (**B**) quantification of LDHA protein expression using band densitometry in OA chondrocytes and chondrocytes from MN cartilage maintained in 18.9% vs. 1% oxygen. (Note: β-actin for this figure is the same as that for Figure 3A since both proteins were stained on the same blot). (**C**) Western blots for HIF1α and (**D**) quantification of HIF1α protein expression using band densitometry in OA chondrocytes and chondrocytes from MN cartilage maintained in 18.9% vs. 1% oxygen. (Note: β-actin for this figure is the same as that for Figure 4E since both proteins were stained on the same blot). Pt = patient. Data shown are mean ± 95% CI. All data were analysed by two-way ANOVA. Statistically significant differences between groups following post hoc testing are shown on graphs. *p* < 0.05 was considered statistically significant.

As LDHA directly catalyses lactate production in glycolysis [29] and hypoxia-inducible factor-1α (HIF1α) promotes glycolytic gene expression in hypoxia [30], we measured protein levels of LDHA and HIF1α by Western blot. Levels of LDHA were overall higher in chondrocytes from both cartilage regions in 1% compared to 18.9% oxygen. There was no difference in LDHA protein levels between chondrocytes from the two cartilage regions in either 18.9% or 1% oxygen (Figure 6A,B). Similarly, HIF1α protein levels were significantly higher in chondrocytes from both cartilage regions in 1% compared to 18.9% oxygen and there was no difference in HIF1α levels between chondrocytes from the two cartilage regions (Figure 6C,D).

### 2.5. Chondrocytes from Macroscopically Normal Cartilage Utilise More Oxidative Phosphorylation

**Figure 7:**
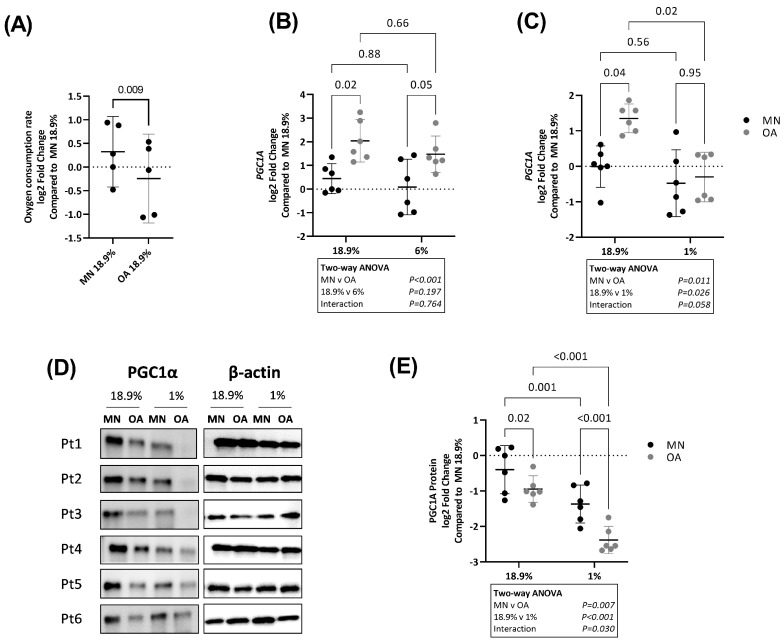
Comparison of oxygen consumption rate and PGC1α expression in OA chondrocytes and chondrocytes from macroscopically normal cartilage maintained in 18.9% compared to 6% or 1% oxygen. Paired samples of human chondrocytes isolated from macroscopically normal (MN) and osteoarthritic (OA) cartilage regions within OA joints were plated in duplicate plates. One plate was transferred to a standard tissue culture incubator (18.9% oxygen) and the other transferred to a hypoxia incubator maintained at either 6% or 1% oxygen for 24 h. Chondrocytes from a different set of patients were used to compare the effects of 18.9% vs. 1% oxygen than those used to compare the effects of 18.9% vs. 6% oxygen. (**A**) Comparison of oxygen consumption rate in OA chondrocytes and chondrocytes from MN cartilage maintained in 18.9% oxygen. Comparison of RNA levels (measured by qPCR) of (**B**) PGC1α in cells maintained in 18.9% vs. 6% oxygen and (**C**) PGC1α in cells maintained in 18.9% vs. 1% oxygen. (**D**) Western blots showing PGC1α protein levels and (**E**) quantification of PGC1α protein expression using band densitometry in OA chondrocytes and chondrocytes from MN cartilage maintained in 18.9% vs. 1% oxygen. Pt = patient. Data shown are mean ± 95% CI. All data were analysed by two-way ANOVA. Statistically significant differences between groups following post hoc testing are shown on graphs. *p* < 0.05 was considered statistically significant.

Next, we assessed oxygen consumption rate (OCR) in OA chondrocytes and chondrocytes from MN cartilage. As expected in 1% oxygen, OCR was negligible, and it was not possible to compare OCR between chondrocytes from the two cartilage regions under this condition. In 18.9% oxygen, OCR was significantly lower in OA chondrocytes compared to chondrocytes from MN cartilage (Figure 7A).

RNA levels of PPARγ co-activator 1α (*PGC1A*), a regulator of mitochondrial biogenesis and mitochondrial energy metabolism [31], were higher in OA chondrocytes compared to chondrocytes from MN cartilage in both 18.9% and 6% oxygen (Figure 7B). In contrast, PGC1α protein levels were lower in OA chondrocytes compared to chondrocytes from MN cartilage in both 18.9% and 1% oxygen (Figure 7D,E). Overall, PGC1A protein levels were lower in chondrocytes from both cartilage regions in 1% compared to 18.9% oxygen (Figure 7D,E).

**Figure 8:**
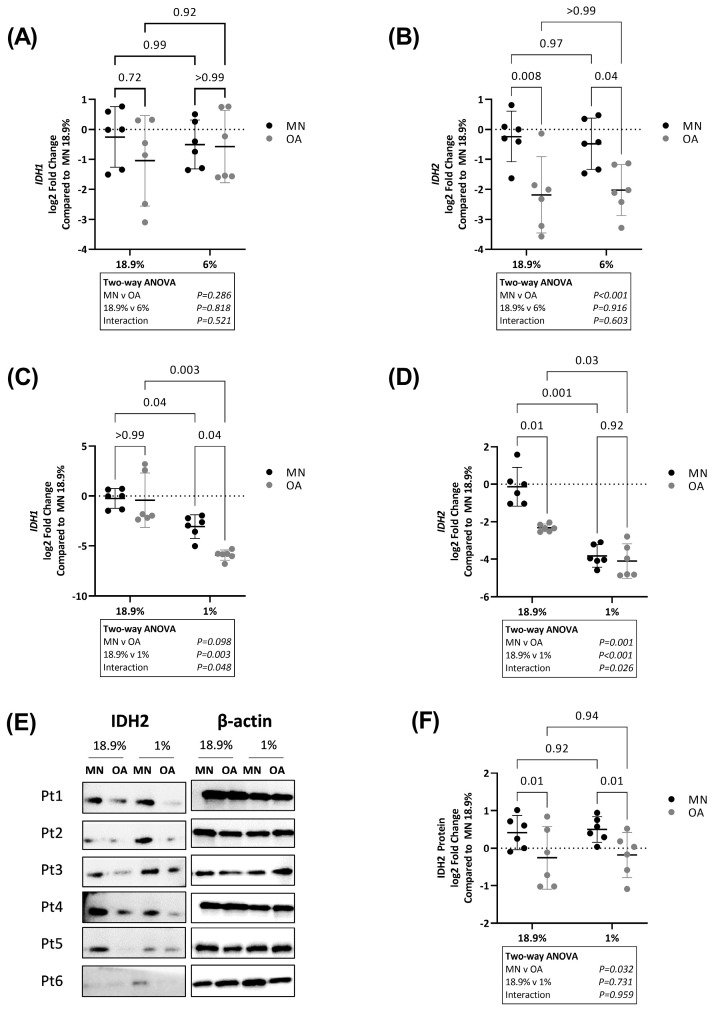
Expression of IDH1 and IDH2 in OA chondrocytes and chondrocytes from macroscopically normal cartilage maintained in 18.9% compared to 6% or 1% oxygen. Paired samples of human chondrocytes isolated from macroscopically normal (MN) and osteoarthritic (OA) cartilage regions within OA joints from six patients were plated in duplicate plates. One plate was transferred to a standard tissue culture incubator (18.9% oxygen) and the other transferred to a hypoxia incubator maintained at either 6% or 1% oxygen for 24 h. Chondrocytes from a different six patients were used to compare the effects of 18.9% vs. 1% oxygen than those used to compare the effects of 18.9% vs. 6% oxygen. Comparison of RNA levels (measured by qPCR) of (**A**) IDH1 and (**B**) IDH2 in cells maintained in 18.9% vs. 6% oxygen and (**C**) IDH1 and (**D**) IDH2 in cells maintained in 18.9% vs. 1% oxygen. (**E**) Western blots showing IDH2 protein levels and (**F**) quantification of IDH2 protein expression using band densitometry in OA chondrocytes and chondrocytes from MN cartilage maintained in 18.9% vs. 1% oxygen. (Note: β-actin for this figure is the same as that for Figure 7D since both proteins were stained on the same blot). Pt = patient. Data shown are mean ± 95% CI. All data were analysed by two-way ANOVA. Statistically significant differences between groups following post hoc testing are shown on graphs. *p* < 0.05 was considered statistically significant.

Next, we analysed expression of *IDH1* and *IDH2*, two hypoxia-sensitive genes encoding enzymes involved in oxidative phosphorylation [29]. Expression of *IDH1* was not significantly different between OA chondrocytes and chondrocytes from MN cartilage in either 18.9% or 6% oxygen (Figure 8A). However, *IDH1* levels were lower in chondrocytes from both cartilage regions when maintained in 1% compared to 18.9% oxygen. The magnitude of the decrease was greater in OA chondrocytes and overall *IDH1* levels were significantly lower in OA chondrocytes compared to chondrocytes from MN cartilage in 1% oxygen.

Similar to *IDH1*, RNA levels of *IDH2* were lower in chondrocytes from both cartilage regions when maintained in 1% oxygen compared to 18.9%. However, the magnitude of the decrease was greatest in chondrocytes from MN cartilage compared to OA chondrocytes and there was no longer a significant difference in *IDH2* expression between chondrocytes from the two cartilage regions in 1% oxygen. In contrast, IDH2 protein levels were lower in OA chondrocytes compared to chondrocytes from MN cartilage at both 18.9% and 1% oxygen (Figure 8E,F) and there was no significant effect of oxygen levels on IDH2 protein levels.

## 3. Discussion

By simultaneously culturing chondrocytes isolated from the same patients under standard tissue culture conditions (18.9% oxygen, hyperoxia) and either 6% (physoxia) or 1% oxygen (hypoxia), this study demonstrated that the nature of the difference in phenotype marker expression and energy metabolism between the two cell populations varies depending on oxygen availability. Although previous studies have examined energy metabolism or phenotype differences under standard tissue culture conditions or hypoxia [24,26,27], to our knowledge, this is the first study to determine the behaviour of human chondrocytes maintained under physoxic conditions and the first study to directly compare behaviour of the same cells maintained under different oxygen levels.

Consistent with previous studies [5,6,10,32], we found OA chondrocytes had higher expression of *ADAMTS5* and *MMP13* and reduced expression of *SOX9* and *ACAN* compared to chondrocytes from MN cartilage when maintained under standard tissue culture conditions. Importantly, we showed that these differences were also apparent between chondrocytes from the two cartilage regions when maintained in 6% oxygen, demonstrating that the same distinct differences in phenotype marker expression observed between OA chondrocytes and chondrocytes from MN cartilage under hyperoxic standard tissue culture conditions are also apparent in physoxia.

Hypoxia has previously been shown to promote new cartilage synthesis and inhibit the expression of cartilage-degrading enzymes by chondrocytes [21,26,27,28,33]. Previous studies have found that *MMP13* and *ADAMTS5* expression are decreased in hypoxia in both OA and normal chondrocytes [26,27]. However, the magnitude of the reduction was found to be considerably greater in OA chondrocytes as both genes were only minimally expressed in normal chondrocytes even under oxygenated conditions [26,27]. In the present study, we also observed reduced *MMP13* and *ADAMTS5* expression in hypoxia in OA chondrocytes, but basal expression of both genes was already very low in chondrocytes from MN cartilage and no further repressive effect of hypoxia was evident on either *MMP13* or *ADAMTS5* in these cells.

Hypoxia has been shown to promote expression of *SOX9, COL2A1* and *ACAN* in rat growth plate chondrocytes, normal human chondrocytes and the C3H10T1/2 cell model of mesenchymal stem cell chondrogenesis [21,30,31,34]. Here, we extend these findings demonstrating that hypoxia increases SOX9, COL2A1 and ACAN protein synthesis by chondrocytes from MN but not OA cartilage. Although we found RNA levels of *SOX9* and *COL2A1* were elevated in chondrocytes from both cartilage regions in hypoxia, this did not translate to an increase in protein levels in OA chondrocytes. This suggests that post-transcriptional control mechanisms may differ between chondrocytes from MN cartilage and OA chondrocytes. In support, protein expression of both SOX9 and COL2A1 is known to be heavily regulated by mechanisms independent of gene transcription [35,36] and post-transcriptional mechanisms have recently been implicated in the differential control of COL2A1 between juvenile and adult chondrocytes [37].

Aside from a difference in phenotype, OA chondrocytes also demonstrate a difference in energy metabolism compared to normal chondrocytes or chondrocytes from adjacent MN cartilage in OA joints [17,24,25,38]. Previous studies have shown that OA chondrocytes maintained under hyperoxic conditions exhibit the Warburg effect, preferentially utilising glycolysis rather than oxidative phosphorylation even when oxygen levels are non-limiting [17,25,38]. Our data build on these findings, demonstrating that OA chondrocytes also utilise glycolysis to a greater extent than chondrocytes from MN cartilage under physoxic conditions. Although the energy yield of glycolysis is low compared to oxidative metabolism, by-products formed during glycolysis also feed into other pathways (e.g., pentose phosphate pathway and Krebs cycle) where they are utilised for nucleotide, lipid and protein synthesis forming essential building blocks for cellular biosynthesis [39]. In contrast, oxidative phosphorylation results in complete catabolism of substrates to carbon dioxide and water. Preferential use of glycolysis is a key mechanism employed by many tumour cells to sustain their high level of proliferative and synthetic activity in disease [39]. The consequences of increased glycolysis for OA have been less well studied. However, inhibition of glycolysis has been shown to reduce synthesis of cartilage-degrading enzymes as well as inflammatory cytokines by OA chondrocytes, indicating that glycolysis facilitates the disease-associated activity of OA chondrocytes [24].

When the oxygen level is limiting, the transcriptional regulator HIF1α is stabilised. HIF1α directly drives glycolytic gene expression (e.g., *LDHA* and *PGK1*), leading to increased glycolysis [30]. Here, we found OA chondrocytes further increased glucose consumption and lactate production in hypoxia, indicating an even greater usage of glycolysis in hypoxic compared to oxygenated conditions. However, the same did not occur in chondrocytes from MN cartilage, indicating these cells maintained a similar level of glycolytic flux under both oxygenated and hypoxic conditions. HIF1α levels were similarly upregulated in chondrocytes from the two cartilage regions in hypoxia. Although RNA levels of HIF1α target genes *GLUT1*, *PGK1* and *LDHA* were lower in chondrocytes from MN compared to OA cartilage in hypoxia, GLUT1 and LDHA proteins were similarly increased in chondrocytes from both cartilage regions. Therefore, the difference in glucose uptake and lactate production between OA chondrocytes and chondrocytes from MN cartilage under hypoxia was not due to a lack of abundance of either the GLUT1 glucose transporter or LDHA.

Interestingly, compared to OA chondrocytes we found chondrocytes from MN cartilage retained higher levels of PGC1α and IDH2 in hypoxia (two proteins involved in mitochondrial biogenesis and oxidative phosphorylation) [29,30]. This is potentially important as even in hypoxia low levels of oxygen are still available, meaning low levels of oxidative phosphorylation are possible [40]. Chondrocytes from MN cartilage have previously been shown to have higher mitochondrial mass, less mitochondrial damage and a greater capacity for oxidative phosphorylation than OA chondrocytes [24]. Our data suggest that some of this increased capacity may also be retained in hypoxia. Although we found OCR was below detectable limits in chondrocytes from both cartilage regions in hypoxia, indicating chondrocytes from MN cartilage were not utilising increased oxidative phosphorylation under our study conditions, oxidative phosphorylation has been shown to be an important contributor to chondrocyte energy metabolism under hypoxic conditions in vivo [40]. Chondrocytes within the hypoxic deep zone of cartilage have been shown to rely on oxygen-dependent pathways to meet some of their energy needs [40]. As these cells are located at some distance from the nutrient-containing synovial fluid, this may serve as an adaptative advantage, enabling substrates other than glucose (e.g., fat) to also be used, and reducing the overall substrate requirement due to the higher energy yield of oxidative phosphorylation [16]. Our findings may mean that chondrocytes from MN cartilage are better equipped than chondrocytes from OA cartilage for survival when glucose levels are limiting.

The difference in response that we observed between OA chondrocytes and chondrocytes from MN cartilage to oxygenated and hypoxic conditions may have significance for understanding the OA disease process. In mice, oxygen levels in cartilage have been shown to increase with OA progression [41]. This is interesting as although human OA cartilage has been proposed to be less oxygenated than normal cartilage, this is based on findings that HIF1α protein levels are higher in OA cartilage [42]. Hypoxia-independent stabilisation of HIF1α has since been demonstrated in OA chondrocytes [43], indicating that HIF1α is not a reliable indicator of hypoxia in OA cartilage. It is plausible that, as in mice, loss of cartilage thickness in human disease could also lead to increased cartilage oxygenation. Our finding that OA chondrocytes exhibit increased catabolic enzyme production under oxygenated conditions indicates that the level of cartilage oxygenation may influence the disease process. Increased tissue oxygenation may promote further tissue catabolism creating a feed-forward mechanism contributing to disease progression. Increased tissue oxygenation may also inhibit the ability of chondrocytes from MN cartilage to produce COL2A1 and ACAN, exacerbating the imbalance between new cartilage synthesis and cartilage breakdown seen in disease [44]. Interestingly, increasing HIF1α stabilisation has been shown to protect against cartilage loss in mice, suggesting that increased activation of hypoxia-triggered pathways may be a potential strategy for OA treatment [45].

A limitation of our study is that we only examined chondrocyte response under a single glucose concentration (5 mM) in monolayer culture. Although this glucose concentration is within the range of normal blood glucose, glucose availability in vivo is likely to vary over time and depend on the relative location of chondrocytes within the 3D cartilage matrix. The impact of glucose availability may be important as increased utilisation of glycolysis by OA chondrocytes means that these cells are dependent on glucose as an energy source since substrates such as fat cannot be metabolised by this pathway [16]. This is interesting in light of evidence that elevated blood glucose/diabetes is a risk factor for OA [46]. It is possible that increased glucose levels facilitate glycolytic energy production in OA chondrocytes, fuelling their disease-associated activity. Determining whether this is the case is of interest as it may provide mechanistic insight into the association between diabetes and OA.

## 4. Materials and Methods

### 4.1. Primary Human Chondrocyte Isolation

Cartilage was collected with informed written consent from patients with osteoarthritis undergoing total knee arthroplasty. Ethical approval was granted by the Health and Disability Ethics Committee, Ministry of Health, New Zealand (approval numbers NTX0506058 and 21CEN191). Tissue was obtained from 20 donors aged 56–85 years, with equal numbers of males and females. All OA joints used in this study were Kellgren–Lawrence grade 4. The modified Mankin scoring system was used to define macroscopically normal (MN) and osteoarthritic (OA) cartilage regions within each joint by the method described in Pauli et al. [47]. MN cartilage was defined as Mankin grade 1 (smooth, non-eroded in appearance). Osteoarthritic cartilage was defined as Mankin grade 8 (fibrillation/cartilage loss to radial zone. This tissue was often adjacent to regions of full thickness cartilage loss, Mankin grade 10). Paired samples of MN and osteoarthritic cartilage from each patient were digested in collagenase (1 mg/mL, 18 h, 37 °C). Isolated chondrocytes were plated at 50,000 cells/mL in DMEM (5 mM glucose) (# 10567022, Thermofisher Scientific, Waltham, CA, USA) with 10% FBS and 1% penicillin/streptomycin. All cells were initially maintained in a standard humidified incubator at 37 °C, 5% CO_2_, 95% air (18.9% oxygen). Cells were allowed to reach 70–80% confluence (approximately 70,000–80,000 cells/mL) before storage in liquid nitrogen. For experiments, cells were revived, allowed to recover in 18.9% oxygen for three days, then plated as described for individual experiments.

### 4.2. Cell Culture

Paired samples of chondrocytes from MN and OA cartilage from 6 patients were seeded at 50,000 cells/mL in duplicate plates. One plate was transferred to a standard tissue culture incubator (18.9% O_2_) and the other to a Heracell™ VIOS 160i Tri-Gas CO_2_ hypoxia incubator (Thermofisher Scientific, Waltham, CA, USA) set at either 6% O_2_ or 1% O_2_. Cell lysates and/or supernatants for subsequent analysis were collected after 24 h using a low-oxygen workstation for the 6% or 1% oxygen conditions (Coy Lab. Products Inc., Grass Lake, MI, USA).

### 4.3. Real Time PCR

cDNA was prepared by a direct cell-to-cDNA method. Briefly, cells were lysed using cell lysis buffer II (Thermo Scientific, Waltham, MA, USA) and DNAase treated before reverse transcription using random primers and MMLV Reverse Transcriptase (Thermo Scientific, Waltham, MA, USA). qPCR was performed on a QuantStudio 12K Flex machine using SYBR Select master mix (both Thermo Scientific, Waltham, MA, USA). Primers are listed in Table S1. Data were analysed using the ΔΔcT method with reference to the housekeeper gene 18S.

### 4.4. Western Blotting

Cell lysates were prepared using RIPA buffer supplemented with sodium orthovanadate, sodium fluoride and phenyl methyl sulphonyl fluoride (1 mM each). Total protein was quantified using the Pierce 660 Protein Assay (Thermo Scientific, Waltham, MA, USA) and equal amounts of protein loaded on to SDS-PAGE gels. Western blotting was carried out using standard protocols. Blots were probed with primary antibodies for HIF1α (ab279654), SOX9 (ab185966), GLUT1 (ab128033), LDHA (ab47010), IDH2 (ab129180) and PGC1α (ab191838) (Abcam, Cambridge, UK).

### 4.5. ELISA

COL2A1, MMP13 and ACAN DuoSet ELISA assays (R&D Systems, Minneapolis, MN, USA) were conducted following the manufacturer’s protocol and corrected for differences in cell number using CyQUANT assay (Thermo Scientific, Waltham, MA, USA).

### 4.6. Glucose Consumption and Lactate Production Assays

Glucose consumption and lactate production were measured using Glucose-Glo and Lactate-Glo assays, respectively (Promega, Madison, WI, USA), following the manufacturer’s protocol. Measurements were corrected for differences in cell number using CyQUANT assay (Thermo Scientific, Waltham, MA, USA).

### 4.7. Oxygen Consumption Rate (OCR)

Paired samples of chondrocytes from MN and OA cartilage were seeded in duplicate plates at a density of 80,000 cells/well (the minimum density that allowed OCR measurement). Plates were incubated for 18 h in 18.9% or 1% oxygen. Following 6 h of serum starvation, cells were allowed to recover for 18 h and OCR measured over a 3 h period using a MitoXpress Xtra O_2_ consumption assay (Agilent Technologies, Santa Clara, CA, USA) and CLARIOstar Plus microplate reader (BMG Labtech, Ortenberg, Germany).

### 4.8. Statistical Analysis

Measurements of all the samples are expressed as the log2 fold change relative to a reference MN sample. The reference MN sample in each case was chosen with the value closest to the mean of all MN sample values. Fold change was calculated as the value of each sample divided by the value of a reference sample, then log2 transformed. Parametric data were analysed by two-way ANOVA with Greenhouse–Geisser correction for non-sphericity and post hoc Tukey testing or by a paired *t*-test when only two variables were compared. Non-parametric data were analysed using a Kruskal–Wallis test with post hoc Dunn’s testing. All data were analysed using GraphPad Prism version 9.0.2. A *p* value of <0.05 was considered statistically significant. Data are presented as mean ± 95% CI.

## Data Availability

The data presented in this study are available in “Differential effects of hypoxia versus hyperoxia or physoxia on phenotype and energy metabolism in human chondrocytes from osteoarthritic compared to MN cartilage” and Supplementary Figure S1.

## Supplementary Materials

The following supporting information can be downloaded at: https://www.mdpi.com/article/10.3390/ijms24087532/s1.
