# Supplementary material for: Differential Effects of Hypoxia versus Hyperoxia or Physoxia on Phenotype and Energy Metabolism in Human Chondrocytes from Osteoarthritic Compared to Macroscopically Normal Cartilage
Source: Int J Mol Sci. 2023 Apr 19;24(8):7532. doi: 10.3390/ijms24087532 (PMC10142591; doi:10.3390/ijms24087532)
Supplement: Supplementary file 1 [file ijms-24-07532-s001.zip › ijms-2353175-supplementary.pdf]

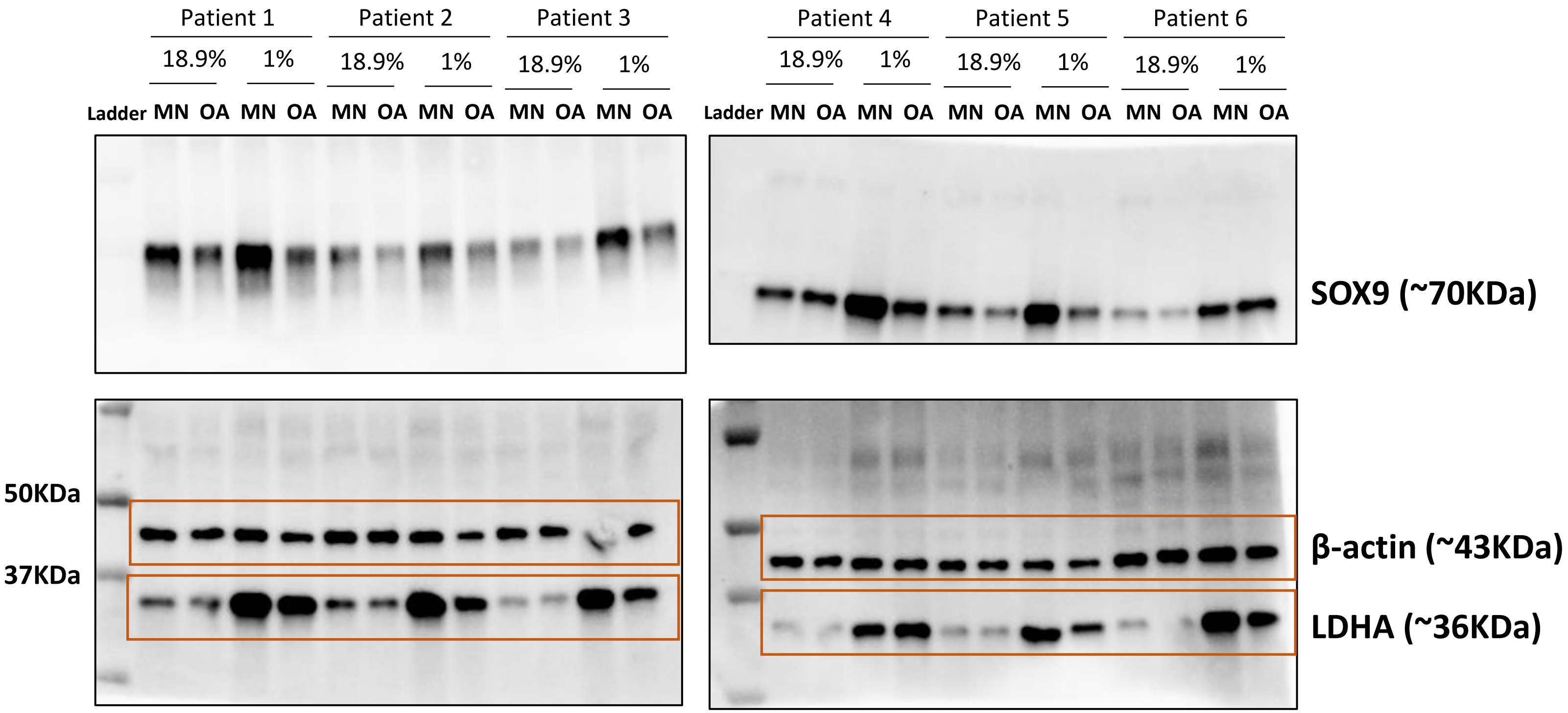

Figure 3A

Figure 6A

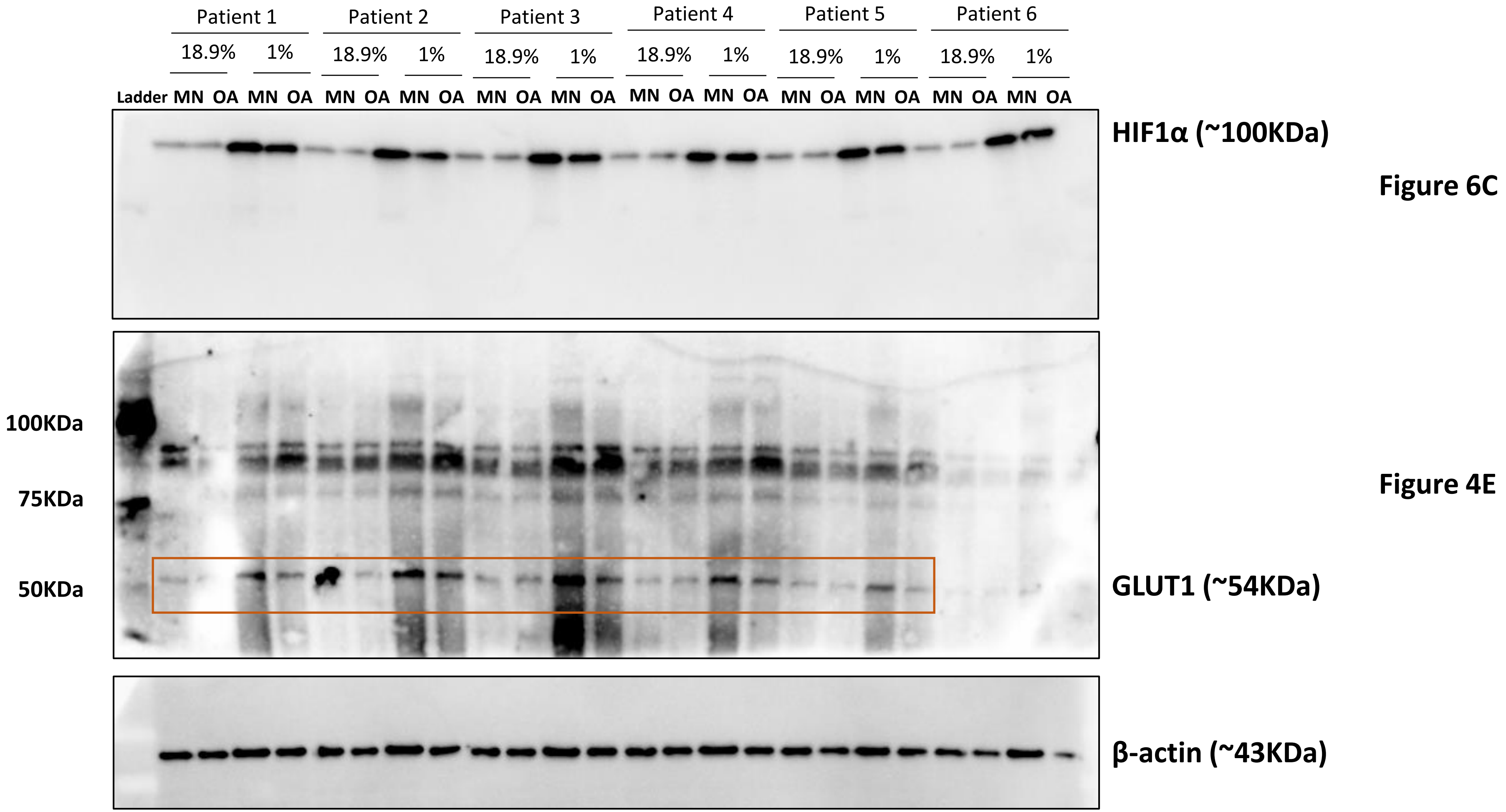

Patient 1
Patient 2
Patient 3
Patient 4
Patient 5
Patient 6
18.9%
1%
Ladder
MN
OA
HIF1α (~100KDa)
Figure 6C
100KDa
75KDa
50KDa
Figure 4E
GLUT1 (~54KDa)
β-actin (~43KDa)

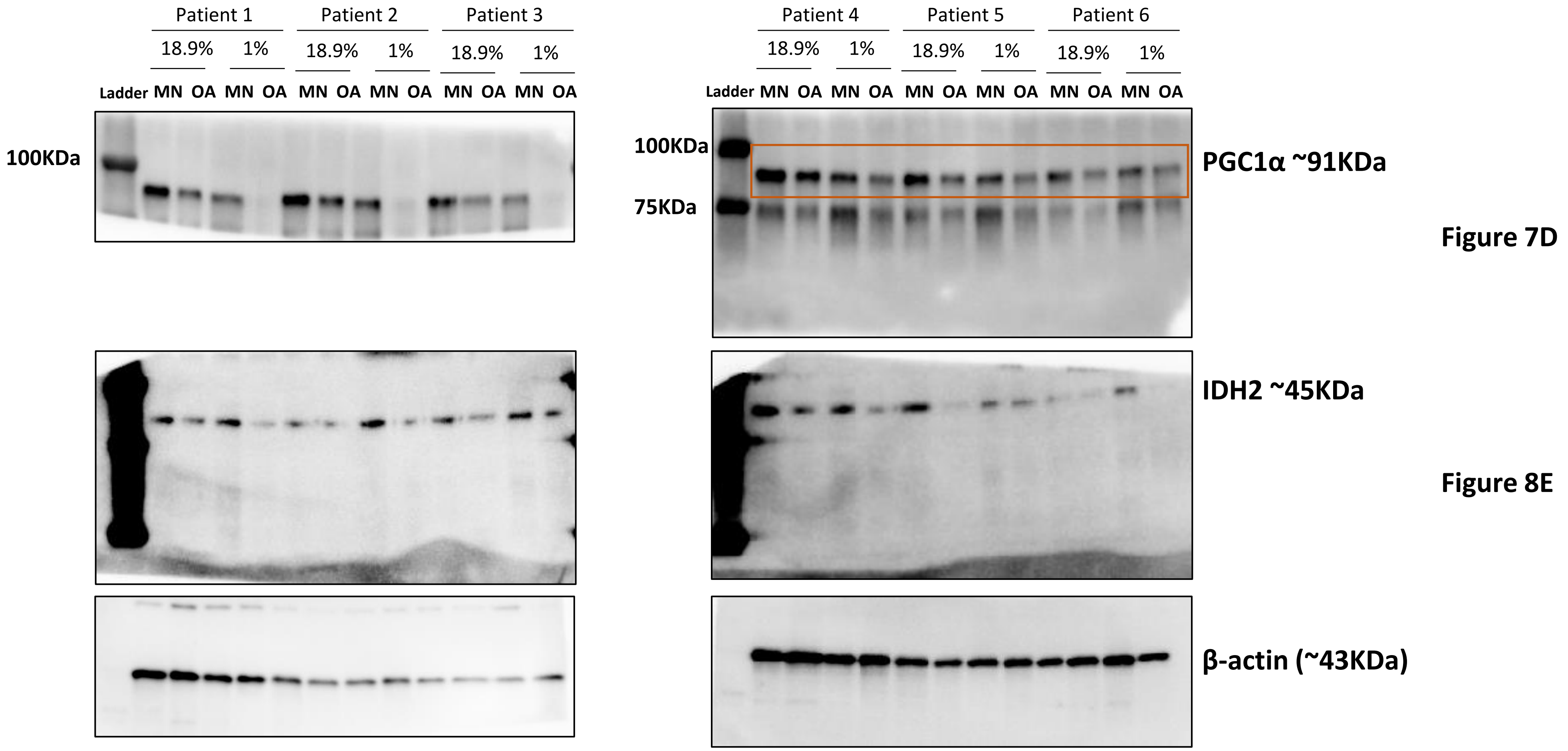

Figure S1. Full length Western blot images.

Table S1 Sequences for primers used for real time PCR.

	Sequence (5'-3')
SOX9	CTG GGC AAG CTC TGG AG<br>CGT TCT TCA CCG ACT TCC TC
COL2A1	CCT CAA GGA TTT CAA GGC AAT<br>GTT TTC CAG CTT CAC CAT CAT C
ACAN	AGA TTC ACA GAA CTC CAG TGC<br>ACC TAC GAT GTC TAC TGC TTT G
MMP13	TTA AGG AGC ATG GCG ACT TCT AC<br>CCC AGG AGG AAA AGC ATG AG
ADAMTS5	CAC TAC GAT GCA GCT ATC CTG<br>AAC ATA TGG TCC CAA CGT CTG
GLUT1	GTG CCA TAC TCA TGA CCA TCG<br>GGC CAC AAA GCC AAA GAT G
LDHA	GAC ATC AGA AGA CTT TAA AAT TGC AG<br>ATG TTG CTG GTG TCT CTC TG
PGK1	GAC AGC AGC CTT AAT CCT CTG<br>CTA ACA AGC TGA CGC TGG A
PGC1α	TGT CTG TAT CCA AGT CGT TCA C<br>GAG TCT GTA TGG AGT GAC ATC G
IDH1	CTG CTT CTA CTG TCT TGC CA<br>ACT ATG ATG GTG ACG TGC AG
IDH2	CCT CAA TCG TCT TCC CAT CAG<br>GCT CAG GTC CTC AAG TCT TC
